# Multinodular and Vacuolating Neuronal Tumors: Imaging Features, Diagnosis, and Management Challenges

**DOI:** 10.3390/diagnostics15030334

**Published:** 2025-01-31

**Authors:** Rosalinda Calandrelli, Carlo Augusto Mallio, Caterina Bernetti, Fabio Pilato

**Affiliations:** 1 Advanced Radiology Center (ARC), Department of Oncological Radiotherapy, and Hematology, Fondazione Policlinico Universitario Agostino Gemelli IRCCS, 00168 Rome, Italy; 2 Research Unit of Diagnostic Imaging and Interventional Radiology, Department of Medicine and Surgery, Università Campus Bio-Medico di Roma, Via Alvaro del Portillo, 21, 00128 Rome, Italy; carloaugustomallio@gmail.com (C.A.M.); c.bernetti@policlinicocampus.it (C.B.); 3 Fondazione Policlinico Universitario Campus Bio-Medico, Via Alvaro del Portillo, 200, 00128 Roma, Italy; f.pilato@policlinicocampus.it; 4 Research Unit of Neurology, Neurophysiology, Neurobiology and Psichiatry, Department of Medicine and Surgery, Università Campus Bio-Medico di Roma, Via Alvaro del Portillo, 21, 00128 Roma, Italy

**Keywords:** multinodular and vacuolating neuronal tumors, MRI, imaging findings, differential diagnosis

## Abstract

**Background/Objectives:** Multinodular and vacuolating neuronal tumors (MNVTs) are a type of recently identified benign neuroepithelial tumor with debated malformative or neoplastic origins. This review summarizes their neuroanatomical localization, imaging, histopathology, immunohistochemistry, and diagnostic challenges. **Methods**: A systematic review of PUBMED/MEDLINE was performed in December 2024. **Results:** Of 118 screened articles, 39 were eligible, covering 299 patients. MNVTs are often asymptomatic “leave me alone” lesions, discovered incidentally, though nonspecific symptoms (59.9%) and seizures (19.7%) are reported. Immunohistochemistry reveals variable profiles, reflecting complex cellular differentiation. The characteristic “bubble-like” MRI pattern along the subcortical ribbon and superficial white matter is a reliable diagnostic feature. Rare cortical involvement and atypical band-like lesions occur. MRI signal intensity varies. Over a mean follow-up of 36 months, lesions were stable or non-recurrent, with only one case of progression. **Conclusion:** MVNT imaging mimics other glioneuronal lesions, but reliable diagnostic MRI features include a “bubble-clustered” appearance, lack of cortical involvement, absence of enhancement, and temporal lobe predominance. Hemodynamic and metabolic properties support the diagnosis. Most lesions remain stable, requiring no treatment. Surgical resection is reserved for cases with uncontrolled seizures or atypical locations where the diagnosis is unclear.

## 1. Introduction

Multinodular and vacuolating neuronal tumors (MVNTs) of the cerebrum have recently been recognized as benign neuroepithelial tumors, classified as WHO Grade I, primarily affecting adults [1]. These tumors are characterized by a distinctive histopathological profile including a mixed cellular composition of immature and incompletely differentiated neuronal cells with stromal vacuolation, suggesting an origin from an aberrant progenitor cell type during development [2,3,4,5]. On the other hand, genetic analyses fail to identify characteristic molecular changes and immunohistochemistry does not reveal typical markers [2,3,4,5,6].

This entity was first described in 2013 by Huse et al. in a small case series of adult patients who presented with focal or complex seizure activity [2]. Since then, several additional case reports and case series have been published, leading to its inclusion in the 2016 updated WHO classification of CNS tumors [1] and its classification under the glioneuronal and neuronal tumor category in the fifth edition of the WHO CNS classification in 2021 [7].

To date, there is ongoing debate on whether these lesions should be classified as neoplastic or malformative/hamartomatous; neuroradiological studies, which show no changes over time, suggest a malformative/hamartomatous nature, while occasionally detected genetic mutations support a neoplastic origin [2,8,9,10].

The majority of MVNT cases are asymptomatic and are discovered incidentally during imaging studies performed for chronic headaches. These lesions typically remain stable in size during follow-up and usually do not require further management leading to their designation as “leave-me-alone” lesions [11,12]. However, a smaller portion of cases may cause symptoms or seizures that are unresponsive to medication and require surgical intervention [2,13]. Thus, the final diagnosis is usually established through one of two methods: (1) a pathologic diagnosis in cases where the tumor is surgically resected or (2) a presumptive clinical-radiological diagnosis, based on its distinctive radiological features and the exclusion of similar lesions that may be considered in the differential diagnosis, for patients who do not undergo surgery.

To date, a total of approximately 300 cases involving supratentorial structures, including those without histopathological confirmation, have been reported. Recently, some authors proposed the term ‘multinodular and vacuolating posterior fossa lesions of unknown significance’ (MV-PLUS) to describe rare cases of infratentorial multinodular lesions [14,15].

This paper aims to provide an updated review of the current literature on MVNTs and offers a structured comprehensive overview of the key imaging findings associated with these rare and indolent brain lesions. It focuses on their neuroanatomical localization, imaging patterns, histopathological features, and immunohistochemical findings. Furthermore, it addresses lesions that pose diagnostic challenges and discusses their implications for management during follow-up.

## 2. Materials and Methods

### Literature Search: Eligibility Criteria and Data Extraction

We searched PUBMED/MEDLINE using the MeSH-term “MVNT” or “Multinodular and Vacuolating Neuronal Tumor”. Revision of the literature was performed independently by two authors and studies were selected by consensus.

The titles, abstracts, and full texts were reviewed to identify potentially eligible articles. Articles were included if they met the following criteria: (1) full-text manuscripts were available in English and (2) they presented case reports or case series describing characteristic radiologic findings sometimes associated with pathology-proven cases of MVNTs. Studies were excluded from our analysis if they were systematic reviews, editor letters, duplicate studies across databases, publications in languages other than English, or if full texts were unavailable.

Finally, the selected papers were included for data extraction and evaluation.

**Figure 1:**
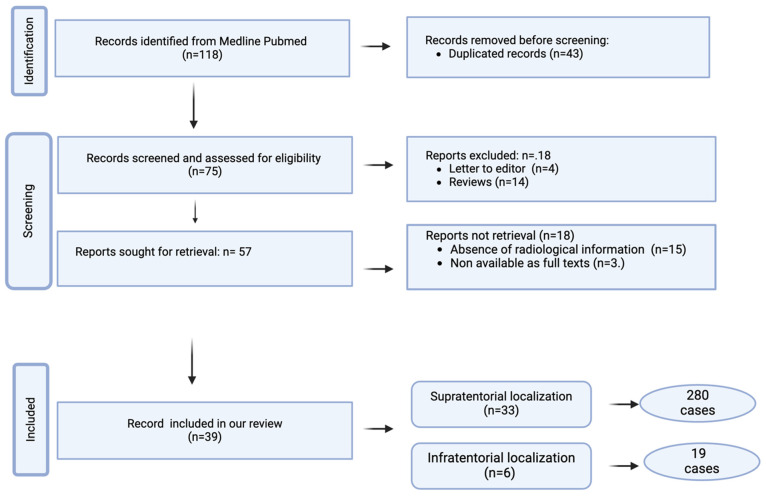
Flow chart of article search and selection.

The following items were extracted from eligible articles: demographics, clinical features, histopathological findings, immunohistochemical findings, molecular/genetic findings, radiological features, treatment, and follow-up. The study selection strategy is shown in Figure 1.

## 3. Results

### Medline Review

We identified 118 articles concerning MVNT. Of these, 43 articles were excluded due to duplication, and an additional 18 were excluded as they were letters to the editor (*n* = 4) or review articles (*n* = 14). Initially, 57 studies were selected, with further exclusions made due to a lack of radiological information (*n* = 15) or unavailability of full-text access (*n* = 3). Ultimately, our review included 39 studies: 33 on supratentorial localization (280 cases) and 6 on infratentorial localization (19 cases). The results are summarized in Table 1.

## 4. Discussion

### 4.1. Demographic Data

The exact epidemiology of these tumors remains unknown, as many cases are likely asymptomatic, incidentally discovered on imaging, or possibly underdiagnosed or misdiagnosed [16]. Although this tumor predominantly develops in adults (with a median age of 43 years, ranging from 21 to 71 years) [10,29], rare pediatric and adolescent cases have been reported (with a median age of 11.5 years, ranging from 6 to 19 years) [8,9,10,11,24,30]. No significant gender predisposition has been observed [20].

### 4.2. Clinical Manifestations

The relationship between MVNT and clinical symptoms remains controversial. The majority of MVNT cases are asymptomatic “leave me alone” lesions, usually discovered incidentally during imaging studies, and do not require any further management [11,16]. Seizures or their equivalents, along with non-focal headaches such as hypoesthesia, loss of postural tone, contact rupture, cognitive impairment, history of migraine, multiple sclerosis, paresthesia, and vertigo, are the most common neurological complaints for which an MRI is requested [21,29].

### 4.3. Histopathologic Features and Immunohistochemical Findings

Histopathologically, MVNTs are characterized by multiple discrete and coalescing islands or nodules of immature neuron-like cells with large eosinophilic vacuolated cytoplasms and prominent round nucleoli [2,3,4,36]. These cells lack a specific orientation and display a ganglionic morphology, with occasional binucleated cells [29]. Among these ambiguous tumor cells, some glial features can also be observed, resembling oligodendroglial and astrocytic cells [6,9,43]; moreover, peri-cellular vacuolation and glial reaction around the nodules may also be present [2,3,4,17].

The nodular islands of aberrant cells, which may abut or merge, are frequently located in the subcortical white matter or within the deeper cortical layers, typically oriented perpendicular to the cortical surface [2,3,4,5]. Cortical involvement is observed in only 10% of MVNT cases and, in areas with cortical nodules, the laminar cytoarchitecture and myeloarchitecture of the cortex remain otherwise intact [3].

MVNTs exhibit a broad and variable immunohistochemical expression profile, reflecting their complex cellular composition and diverse patterns of differentiation. Specifically, tumor cells in MVNTs variably express both immature neuronal markers, such as Human antigen C/Human antigen D (HuC/HuD), alpha-internexin (α-INA), and mature neuronal differentiation markers including Neuronal Nuclei (NeuN), neurofilaments, synaptophysin, and Microtubule-Associated Protein 2 (MAP2) [2,8,10]. Astroglial differentiation markers like Glial Fibrillary Acidic Protein (GFAP) and Glial Fibrillary Acidic Protein Delta Isoform (GFAPδ) may also be expressed, alongside markers for oligodendroglial lineage and myelination such as Oligodendrocyte Transcription Factor 2 (Olig2) and SMI94/myelin basic protein (MBP) [2,10,29]. An extended immunohistochemical panel may reveal additional markers including those specific to cortical layers (TBR1, TBR2, OTX1, N200, MAP1B) and markers of developmental or stem cell characteristics (CD34, Reelin, PAX6, SOX2, Nestin, DCX, PDGFRβ) [10]. Certain markers for interneuronal subsets (such as calbindin, calretinin, parvalbumin, and NPY), chloride co-transporters (NKCC1, NKCC2), and neurodegenerative markers (p62, AT8, APP, mitochondria) may also be detected [10]. The Ki-67 proliferation index is relatively low, suggesting an inert biological behavior [11]. To date, genetic analyses have not identified characteristic oncogenic mutations. However, MVNTs may occasionally exhibit variable mutations in genes related to the MAPK pathway and BRAF or FGFR genes [2,9]. These mutations are primarily small indels and hotspot mutations [9,33]. Although debate is still active regarding whether these lesions are neoplastic or malformative, the presence of genetic alterations supports a neoplastic origin [2,9] (Table 2).

### 4.4. Radiological Findings

#### 4.4.1. Localization and Lesion’s Appearance

The primary site of tumor onset is focused in the cerebral hemispheres, most commonly affecting the temporal and parietal lobes, with less frequent involvement of the frontal and occipital lobes [2,11]. The tumors do not exhibit specific laterality [2,11]. Rare supratentorial cases have been reported in the thalamus, hippocampus, and parahippocampal gyrus [23,27,32,42]. Additionally, only a few cases have been identified in infratentorial regions, specifically occurring in the vermian-paravermian region or the paravermian region with cerebellar hemisphere extension, although not all of these have been pathologically confirmed [14,39,40,41]. Recently, Lecler et al. introduced the term “multinodular and vacuolating posterior fossa lesions of unknown significance” (MV-PLUS) to describe these rare infratentorial occurrences [28].

On CT scans, the lesions are often not detected [6]. However, in some cases, as described by Nagaishi et al. and Osborn et al., the lesions can be detected as a non-calcified non-enhancing hypoattenuating abnormalities in the subcortical white matter [4,11,13].

In contrast, the appearance of these lesions on MRI is distinctive and diagnostic, characterized by clusters of multiple round or ovoid intra-axial nodules, ranging from 1 to 5 mm in diameter. These nodules exhibit a bubble-like appearance and occasionally contain satellite nodules [21,32]. The lesions are located along the subcortical ribbon and the superficial subcortical white matter, following the gyral contour [10,31]. Cortical involvement has been observed in only 10% of MVNT [38]. Although small coalescing nodules may sometimes mimic a mass-like lesion, these lesions are not tumefactive [2].

#### 4.4.2. MRI Signal Intensity

By combining the signals on T1, T2, and FLAIR sequences, the signal intensity of supratentorial nodules is variable. This non-homogeneous appearance depends on the size of the nodules, their organization in clusters, and the extent of cellular and pericellular vacuolization.

The lesions may appear iso- to hypointense relative to the cortex on T1-weighted images and hyperintense to markedly hyperintense on T2-weighted images, with partial, absent, or complete suppression on FLAIR sequences [13,17]. FLAIR sequences, if acquired with thin slices, can help to distinguish both clustered and scattered nodules, which display distinct characteristics based on their size. Particularly, larger nodules exhibit a hypointense center surrounded by a hyperintense rim, resulting in a ring-like appearance, with high-signal-intensity areas connecting the nodules. Smaller nodules demonstrate a non-homogeneous high signal intensity with a central dot of low signal intensity [13,16].

Some authors have suggested the presence of a central cystic component in cases with a suppression pattern on FLAIR imaging [16]. However, this hypothesis has been challenged by others who have reported cases of cystic components also in tumors with a non-suppression pattern on FLAIR images. This feature has been attributed to high protein content within the vacuoles [11]. The periphery of the nodules may show high signal intensity on FLAIR images, which has been attributed to high protein content and neuronal demyelination [13]. Additionally, the areas surrounding the nodules may appear normal [17] or may exhibit high signal intensity on FLAIR images, likely due to the presence of unmyelinated nerve fibers [13].

Some MVNTs may exhibit hyperintense T2-FLAIR extensions toward the ventricle; however, unlike the band-like extensions seen in focal cortical dysplasia (FCD) IIB, these present as bubbly signal alterations [16].

The lesions do not exhibit blooming on SWI, indicating the absence of intratumoral hemorrhage or calcification. Additionally, they do not show restriction on DWI [26]; however, a bright diffusion sign for MVNT diagnosis has been reported, without corresponding low ADC values [11,28,37,38]. Typically, these lesions do not display contrast enhancement, although some cases of weak and focal enhancement have been reported [2]. Notably, Alsufayan et al. observed contrast enhancement in a dotted and linear pattern [16].

**Figure 2:**
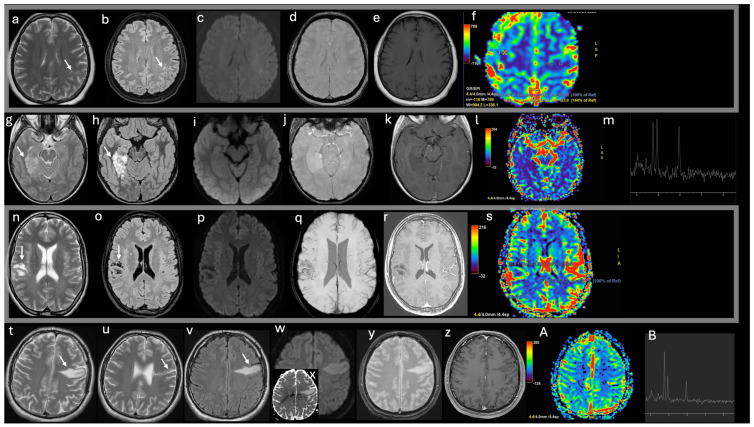
Imaging features of typical and atypical supratentorial MVNT cases. T2 weighted (w) axial images (**a**,**g**,**n**,**t**,**u**), FLAIR axial images (**b**,**h**,**o**,**v**), DWI (**c**,**i**,**p**,**w**), ADC (**x**), T2* w (**d**,**j**,**q**,**y**) images, post-contrast T1w (**e**,**k**,**r**,**z**) images, DSC-CBV maps (**f**,**l**,**s**,**A**), spectroscopy (**m**,**B**).

The findings of advanced MRI techniques in MVNT are limited, poorly understood, and often contradictory, making it challenging to draw general conclusions. Perfusion-weighted imaging (PWI), which provides insights into neoangiogenesis, has yielded mixed findings. Lecler et al. emphasized that hyperperfusion is not observed in these lesions [15]; Gokce and Makrakis reported a slight increase in perfusion, reflecting the absence of significant microvascular proliferation [19,25]; conversely, other studies have noted slightly decreased CBV values [17,25]. Magnetic resonance spectroscopy (MRS), which assesses metabolite concentrations, has also shown variable results. Some studies have reported a mild increase in the choline/creatine and choline/NAA ratios, potentially reflecting tissue disorganization and the presence of dysplastic immature cells [4,13]. Other reports, however, found no choline peak and observed a slight decrease in NAA levels [15] (Figure 2).

- A 60-year-old female patient undergoing follow-up for pulmonary adenocarcinoma (a–f). MRI T2w and FLAIR sequences reveal several tiny hyperintense nodules clustered within the subcortical left parietal white matter, with sparing of the overlying cortex (white arrows in a–b). No restricted diffusion (c) and no intratumoral hemorrhage (d) are observed; post-contrast imaging shows no enhancement (e) and no increased rCBV values (f). The presumed MVNT remained stable during the 2-year follow-up period;
- A 13-year-old male with epilepsy underwent evaluation and was subsequently treated with surgery (g–m). Imaging reveals clusters of coalescent nodules involving the cortex and subcortical white matter in the right parahippocampal gyrus, extending to the hippocampus without tissue expansion or mass effect. The nodules are hyperintense on T2WI and FLAIR, with interspersed areas of high signal intensity (white arrows in g,h). No diffusion restriction (i), hemorrhage (j), enhancement (k), or perfusion alterations (l) are observed. Magnetic resonance spectroscopy shows an elevated choline peak, reduced N-acetyl aspartate peak, and an increased Cho/NAA ratio (m);
- A 20-year-old female with Noonan syndrome (n–s). Imaging reveals nodules with a bubble-like appearance in the subcortical parietal white matter, hyperintense to CSF on T2 (white arrow in n), with complete suppression on FLAIR except for a peripheral high-signal-intensity ring (white arrow in o), following the gyral contour. No vasogenic edema, mass effect, diffusion restriction (p), hemorrhage (q), enhancement (r), or perfusion alterations (s) are observed. The imaging remained stable over the extended 5-year follow-up period;
- A 26-year-old syndromic male with epilepsy was treated pharmacologically (t–B). T2WI and FLAIR imaging reveal a radial-like band of tiny hyperintense nodules clustered in the subcortical-juxtacortical left frontal lobe, extending to the lateral ventricle, with no cortical blurring (white arrows in t,u,v). A bright diffusion signal restriction (w) with high ADC values is detected (y); no intratumoral hemorrhage (y), no enhancement (z), and no perfusion alterations are observed (A). Spectroscopy demonstrates an increased Cho/NAA ratio (B). The imaging remained stable over the extended 6-year follow-up period.

**Figure 3:**
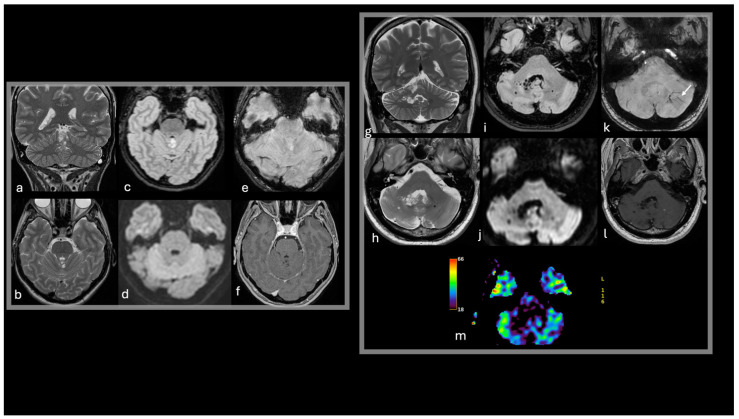
Imaging features suggestive of infratentorial MVNT. T2w coronal images (**a**,**g**), T2w axial images (**b**,**h**), FLAIR axial images (**c**,**i**), DWI (**d**,**j**), SWI (**e**,**k**), post-contrast T1w (**f**,**l**), DSC-CBV map (**m**).

The uncommon infratentorial MVNT/MV-PLUS lesions are often characterized by cystic (or cyst-like) nodular formations frequently associated with cortical involvement [14,15]. These lesions typically appear hypointense on T1-weighted images, while T2-weighted and FLAIR sequences reveal hyperintense or isointense signals relative to normal white matter. In some cases, fuzzy ripple-like changes and cystic features are observed with suppression or partial suppression on FLAIR imaging [14,15,40]. Magnetic resonance spectroscopy in these rare cases does not indicate significant alterations of major brain metabolites, while perfusion-weighted imaging (when performed) demonstrates hypoperfused lesions compared to normal cerebellar tissue, further supporting their benign and non-aggressive nature [14] (Figure 3).

- A 52-year-old female presenting with headaches and an incidental finding of small coalescent T2w and FLAIR hyperintense nodules in the superior vermis (a,b,c), without diffusion restriction (d), intratumoral susceptibility signals (e), enhancement (f), or mass effect. The imaging remained stable during the 3-year follow-up period;
- A 57-year-old male presented with speech disturbances and an incidental finding of small nodules in the right paravermian region and cerebellar hemisphere. The nodules are hyperintense to CSF on T2 (g,h) with FLAIR suppression, except for a peripheral high-signal-intensity ring (i). Abnormal venous drainage is noted in the left cerebellar hemisphere (white arrow in k). No diffusion restriction (j), intratumoral susceptibility signals (k), enhancement (l), or perfusion alterations (m) are observed. The imaging remained stable during the 2-year follow-up period.

When accurately diagnosed, follow-up MRI examinations have shown no changes in lesion size or signal characteristics over time.

### 4.5. Treatment and Follow-Up

When a tumor is discovered incidentally during imaging studies, but the patient has no symptoms related to the tumor, careful monitoring is the most appropriate approach. A number of case reports reporting follow-up MRI studies showed radiological stability of the lesions [10,31,34]. Volumetric assessments of MVNTs across serial follow-up brain MR imaging examinations have shown no significant changes in either absolute or percentage volume compared to baseline scans [34]. Stable radiological follow-up confirms that a definitive diagnosis could rely only on imaging criteria and that pathological confirmation is often not mandatory.

Conversely, when the patient shows epilepsy, therapeutic management can be pursued with either antiepileptic drugs or surgery [2,13,22]. Drug therapy offers the advantage of being non-invasive, though it comes with the potential for side effects and requires lifelong administration [13]. Surgical intervention, on the other hand, provides the opportunity for complete seizure remission and allows for histological analysis to confirm the diagnosis, which was previously only probable based on imaging criteria [2,13]. However, surgery also carries potential risks, including postoperative seizures or neurological deficits related to the surgical site [13].

### 4.6. Differential Diagnosis

The most common supratentorial differential diagnoses include low-grade gliomas (LGG), gangliocytoma, and enlarged Virchow-Robin perivascular spaces (VRPSs) in adults. In children and adolescents, the most frequent differential diagnoses are ganglioglioma, FCD IIB, and disembryoplastic neuroepithelial tumor (DNET).

DNETs are the tumors that more commonly mimic MVNTs. They primarily occur in children and adolescents and are rare in older individuals [35]. DNETs are often associated with epilepsy and occasionally with focal cortical dysplasia [26]. Imaging typically reveals multiple nodules with a characteristic “specific glioneuronal element,” featuring a columnar arrangement of mature dysplasia-free oligodendroglia-like cells (OLCs) floating within a mucinous lake [17]. Immunohistochemical staining shows NeuN-positive neurons and upregulation of microglial markers [29]. DNETs are usually located in the cortical regions, particularly the temporal and frontal lobes [44]. They often present as thickened cerebral cortexes with a well-demarcated wedge-shaped lobulated appearance and a characteristic bubbly appearance, without significant mass effect or edema. On FLAIR-weighted imaging, DNETs show mixed-signal intensity with some cyst-like components suppressed and a surrounding bright rim [32]. About one-third show enhancement on post-contrast imaging [17,26]. Calcifications and hemosiderin staining are relatively common, and on CT scans, they may cause scalloping of the skull’s inner table in about 44–60% of cases [10].

Imaging differences between MVNTs and DNETs include MVNTs’ lack of cortical involvement in most cases, their consistent location in the deep white matter, and the distinctive clustering of nodules with a “bubble” appearance.

FCD IIB, a common cause of chronic refractory epilepsy in children, adolescents, and young adults, is characterized by cortical thickening, blurring of the grey-white matter interface, and, occasionally, abnormal cortical gyration, most often involving the frontal and temporal lobes [16].

Immunohistochemically, FCD IIB features disordered neuronal arrangement with positive NeuN staining, strong GFAP expression, and increased levels of neuronal markers such as SMI-32 [17,45,46]. A hallmark imaging feature is the FLAIR trans-mantle sign—a radial band extending from the cortex, through the affected gyrus, to the lateral ventricle, thinning at a triangular tip pointed toward the ventricle [11,47]. Cortical or subcortical high-signal shadows may also be observed [40]. While MVNTs can occasionally show a FLAIR hyperintense band-like lesion extending radially toward the ventricle, these lesions differ from those in FCD IIB. In MVNTs, the signal alteration appears bubbly rather than strictly band-like, reflecting the distinctive nodular structure of MVNTs [16].

*VRPSs* are incidental findings observed in up to 13% of healthy adults and are less commonly seen in children [48]. They are most often found in regions with numerous perforating vessels, appearing elongated along the vessel axis and located near subarachnoid spaces. Common sites include the mesencephalo-thalamic region (types 1, 2, and 3) and the subcortical white matter of the anterior superior temporal gyrus (type 4) [49,50,51]. VRPSs typically present as isolated, linear, or fusiform foci with a symmetric distribution. They may occasionally exhibit a bubble-like appearance, resembling MVNTs, but they consistently display CSF-like signals on all MRI sequences [49].

Gangliocytoma/ganglioglioma. Gangliocytomas are more commonly found in adolescents and young adults, whereas gangliogliomas are most prevalent in children and young adults under the age of 30 [52,53]. Both tumor types are characterized by dysmorphic ganglion cells arranged in architectural disarray, often accompanied by inflammation, with gangliogliomas also containing neoplastic glial components [17]. Despite their dysplastic elements, gangliocytomas and gangliogliomas exhibit a mature neuronal immunophenotype, highlighted by NeuN positivity [54].

Although more common in gangliogliomas, both tumor types may exhibit the BRAF V600E mutation, CD34 positivity, and associated features such as eosinophilic granular bodies, Rosenthal fibers, and lymphocytic infiltration [17]. These tumors are typically located in the temporal lobe cortex and often contain solid components that may enhance contrast-enhanced imaging and occasionally calcify. Contrast enhancement and mixed tissue components are more indicative of ganglioglioma than gangliocytoma [17,18]. In cases where cystic features are predominant, sometimes with a nodular or multinodular appearance, they may resemble MVNTs [17].

**Figure 4:**
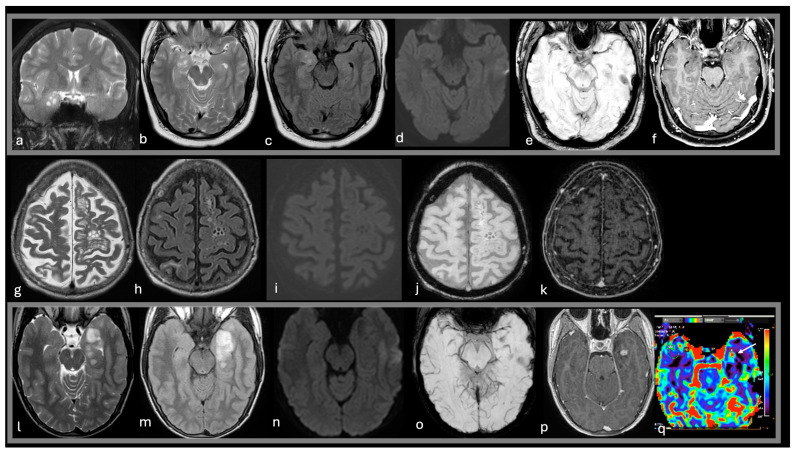
Common lesions in the differential diagnoses of MVNT in the supratentorial compartment. T2w coronal images (**a**), T2w axial images (**b**,**g**,**l**), FLAIR axial images (**c**,**h**,**m**), DWI (**d**,**i**,**n**), SWI (**e**,**j**,**o**), post-contrast T1w (**f**,**k**,**p**), DSC-CBV map (**q**).

LGG, particularly oligodendrogliomas with neuronal differentiation, are more common in adults, with a predilection for the temporal and frontal lobes [55]. Immunohistochemically, LGGs may show OLIG2 positivity, although this marker is not specific for LGGs [29]. These tumors typically present as white matter lesions that extend into the overlying cortex, causing mass effect with minimal contrast enhancement. The cystic components and poor post-contrast enhancement can complicate the differential diagnosis; however, the presence of infiltrative neoplastic glial cells and the tumor’s infiltrative behavior can help to distinguish LGGs from MVNTs [17] (Table 3, Figure 4).

- A 25-year-old male with epilepsy was treated surgically with a histological diagnosis of DNET (a–f). Imaging reveals a shaped, multicystic (bubbly) cortical-subcortical lesion expanding the right temporal uncus (a,b,c). The nodules are hyperintense on T2WI and FLAIR (a,b,c), with no intratumoral diffusion restriction (d), susceptibility signals (e), or enhancement (f).

An 84-year-old male on dialysis underwent imaging due to worsening neurological conditions (g–k). T2WI MRI reveals clusters of variable-sized cysts accompanying penetrating arteries in the left centrum semiovale. Fluid-filled spaces resembling CSF on both T2WI and FLAIR are surrounded by a glial reaction (g,h). No adjacent brain edema, diffusion restriction (i), intratumoral susceptibility signals (j), or enhancement (k) is detected. The presumed diagnosis was enlarged Virchow-Robin perivascular spaces or MVNT, which remained stable during the 2-year follow-up period.

A 15-year-old male with a long history of temporal lobe epilepsy was treated surgically with a histological diagnosis of ganglioglioma (l–q). Imaging shows a cystic and solid temporal lobe mass expanding the overlying cortex, hyperintense on T2WI (l) and FLAIR (m), with blooming areas on SWI (o) and intense enhancement of the mural nodule (p) with focal increased perfusion (arrow in q). No edema or mass effect is noted.

The most common infratentorial differential diagnoses include Rosette-forming glioneuronal tumors (RGNT), pilocytic astrocytomas (PA), dysplastic cerebellar gangliocytomas (DCG), and VRPS.

RGNTs are rare mixed glioneuronal tumors that predominantly affect young individuals, with a male predominance [56]. These tumors consist of two distinct histological components: rosette-like structures surrounding neuropil and blood vessels, expressing synaptophysin, and glial cells with pilocytic or oligodendroglial morphology, expressing Olig2, GFAP, and S-100 [57]. Primary molecular alterations include FGFR1 and PIK3R1 mutations [58,59]. RGNTs typically arise in the midline, affecting the fourth ventricle and cerebellar vermis [60], but can rarely occur in the tectal, pineal, pontine, and thalamic regions [61,62,63,64]. RGNTs are well-circumscribed and may present as solid, cystic-solid, or multi-cystic masses [65,66], the latter resembling MVNTs [67]. On MRI, RGNTs are iso- or hypointense on T1-weighted images and hyperintense on T2-weighted images, with cystic component suppression on FLAIR images. They often show focal contrast enhancement, which can be nodular, linear, ring-like, or spot-like [65]. A characteristic “green bell pepper sign” may be seen after injection of gadolinium-based contrast agents, where the tumor can show a ring of enhancement with central hypointensity, resembling the cross-section of a green bell pepper. This appearance is due to the mucous content of the tumor, which does not enhance at the center, while there is ring enhancement of the surrounding solid components [56]. Additional features may include intratumoral hemorrhage, calcification, CSF dissemination, and multiple satellite lesions.

PAs are slow-growing well-circumscribed WHO grade 1 glial tumors, most commonly found in the cerebellum of children and adolescents, with rare occurrence in the supratentorial compartment of adults [68]. Approximately 90% of cases show abnormalities in the MAPK pathway [68,69]. Histologically, PAs exhibit low-to-moderate cellularity with compact fibrillated areas containing cells with long hair-like (pilocytic) processes, alongside more loosely textured regions of multipolar cells. A biphasic pattern may develop with an additional oligodendroglial component. Immunohistochemistry is typically positive for GFAP, OLIG2, and S-100, with a Ki-67 index of 1–5%. Aggressive behavior is rare but should be considered in cases involving adults or patients with neurofibromatosis type 1 (NF1) [68]. On MRI, pilocytic astrocytomas typically present with a large cystic component and an enhancing mural nodule in about two-thirds of the cases. The solid component usually appears hypointense on T1-weighted images and hyperintense on T2-weighted images, with homogeneous contrast enhancement [70,71]. In rare cases, the tumor may appear multicystic with limited or no enhancement, similar to the MVNT [72].

**Figure 5:**
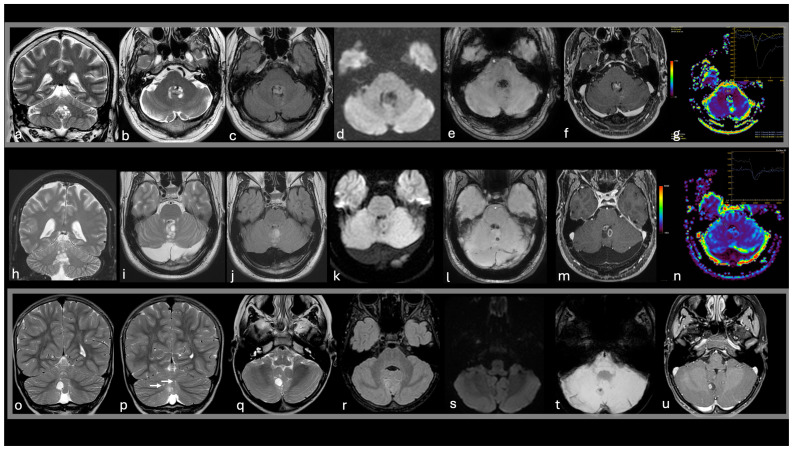
Differential diagnoses of MVNT in the infratentorial compartment. T2w coronal images (**a**,**h**,**o**,**p**), T2w axial images (**b**,**i**,**q**), FLAIR axial images (**c**,**j**,**r**), DWI (**d**,**k**,**s**), SWI (**e**,**l**,**t**), post-contrast T1w (**f**,**m**,**u**), DSC-CBV maps/time curves (**g**,**n**).

DCG, a neuronal and mixed neuronal-glial tumor, primarily affects young adults and is rare in children. Microscopically, it consists of disorganized dysplastic ganglion cells, sometimes associated with irregularly arranged glial cells, with a lack of normal Purkinje cells. These cells are immune-positive for GFAP, S-100, NeuN, and synaptophysin, with a low Ki-67 proliferation index. DCG is often associated with PTEN gene mutations [73]. Upon imaging, DCG appears as a well-defined cerebellar mass with a characteristic ‘tiger-striped’ pattern on T2-weighted MRI, featuring alternating hypointense and hyperintense bands due to thickened cerebellar folia lacking secondary arborization. Occasionally, rounded coalescent ‘cystic’ areas may be present due to pronounced vacuolization [74,75], sometimes mimicking the MVNT. Enhancement is variable, ranging from faint striated to intense patterns, and small intratumoral calcifications may rarely be observed (Table 2, Figure 5).

- A 44-year-old male presenting with headache and vertigo with a histological diagnosis of RGNT (a–g). Imaging shows a solid-cystic mass in the fourth ventricle, hyperintense on T2WI and FLAIR (a,b,c), hypointense on DWI (d), with blooming on SWI (e) and heterogeneous enhancement after gadolinium (f). Perfusion mapping reveals increased rCBV values with reduced T2 signal recovery on the time curve (g);
- A 29-year-old male presenting with headache and vomiting, with a histological diagnosis of RGNT (h–n). Imaging reveals a multicystic mass in the cerebellar vermis extending into the fourth ventricle. The mass is hyperintense on T2WI and FLAIR (h,i,j), hypointense on DWI (k), with tiny blooming foci on SWI (l) and a ’green bell pepper’ sign after gadolinium enhancement (m). Perfusion mapping shows increased rCBV values with reduced T2 signal recovery on the time curve (n);
- A 6-year-old male presenting with a critical episode and histological diagnosis of pylocityc astrocytomas (o–u). Imaging reveals a cystic mass in the cerebellar vermis, consisting of a larger cyst (o,q) with smaller satellite cysts hyperintense to CSF on T2WI (white arrows in p) and unsuppressed on FLAIR (r) without restriction on DWI (s). The larger cyst features blooming foci on SWI (t) and an enhancing mural nodule (u).

## 5. Conclusions

The origin of MVNT, either malformative or neoplastic, remains a topic of debate.

Imaging findings of MVNT can sometimes mimic other tumor-like glioneuronal lesions but certain MRI features, such as the “bubble-clustered” appearance in subcortical white matter, lack of cortical involvement, absence of enhancement, and the predominance of the temporal lobe location, are reliable signs for diagnosing MVNT. Additionally, the lesion’s hemodynamic and metabolic properties support its benign nature. In rare cases of subtentorial MVNT, the diagnosis is less clear due to cortical involvement. However, in asymptomatic cases, strict follow-up with imaging to monitor the lesion’s size and pattern is usually sufficient for confirming the diagnosis and guiding the management.

There are still controversies regarding epilepsy associated with MVNT, as there is ongoing debate over the optimal treatment approach, whether with antiepileptic drugs or surgery. While surgical resection often eliminates seizures without tumor regrowth, it is typically applied for cases with uncontrolled seizures or for lesions in atypical locations where the diagnosis remains uncertain.

## Figures and Tables

**Table 1:** Summary of key articles on clinical, immunohistochemical, genetic, and MRI findings in MVNT.

Authors (Year)	Study Type (n. pts)	Age in Years (Range)	Clinical Presentation	Immunohistochemical Findings/ Genetic Alterations	Localization	MRI Findings	Follow-Up (n.pts) (Months)
Huse et al. (2013) [2]	Case series (n.10)	(31–64)	Seizure (n.3), confusion/loss of attention/vertigo (n.7)	-HuC/HuD, Syn (n.3), CD34 (n.7)/MEK1 (n.1)	Temporal (n.8), frontal (n.1), parietal (n.1)	Appearance: multinodularity and solid (n.9), cystic components (n.1). Signal: T2w/FLAIR hyperintensity (n.10), faint enhancement (n.2), increased choline/NAA (n.1)	No recurrence after surgery (n.10); (8–72)
Bodi et al. (2014) [3]	Case series (n.2)	34–71	Seizure (n.2)	Syn, HuC/HuD, p62	Temporal (n.1), frontal (n.1),	Appearance: multinodularity and solid Signal: T2-w/FLAIR hyperintensity, no enhancement	No recurrence after surgery (n.2); (24)
Fukushima et al. (2015) [5]	Case report (n.1)	37	Epilepsy	Syn, HuC/HuD, Olig2	Parietal	Appearance: solid lesion Signal: T1w isointensity, T2w/FLAIR hyperintensity, no enhancement, increased choline/NAA	No recurrence after surgery (n.1); (18)
Nagaishi et al. (2015) [4]	Case report (n.1)	22	Headache	α-INA, HuC/HuD, Olig2, S100	Frontal	Appearance: solid lesion Signal: T1w isointensity, T2-w/FLAIR hyperintensity, no enhancement, increased choline/NAA	S (n.1); (6)
Yamaguchi et al. (2016) [6]	Case report (n.1)	41	Epilepsy	α-INA, HuC/HuD, Olig2, p62	Temporal	Appearance: multinodular-solid lesion Signal: T1w hypointensity, T2w/FLAIR hyperintensity, no enhancement	Surgery (n.1); (NA)
Nunes et al. (2017) [11]	Case series (n.33)	8–63	Suspected seizures (n.8), headache (n.16)	NA	Parietal (n.9), frontal (n.8), temporal (n.6), occipital (n.2), more lobes (n.8)	Appearance: multinodularity Signal: T2w/FLAIR hyperintensity, T1w iso/hypointensity, no restricted diffusion (n.33), enhancement (n.1)	S (n.30), NA (n.3); (24–144)
Alsufayan et al. (2017) [16]	Original research (n.24)	24–59	Headaches (n.8), seizures (n.4), visual symptom (n.3), paresthesia (n.1), hemiparesis (n.1), cognitive difficulties (n.1), non relevant neurological symptoms (n.6)	NA	Parietal (n.5), frontal (n.11), temporal (n.2), occipital (n.3), more lobes (n.3)	Appearance: multinodularity Signal: a) T2w/FLAIR hyperintensity, T1w hypointensity (solid lesions n.18); b) T2w hyperintensity, suppressed on FLAIR, T1w hypointensity (cystic-solid lesions n.5, cystic lesion n.1) No restricted diffusion (n.24), enhancement (n.2)	S (n.18); (2–93) Progression (n.1) NA (n.5)
Thom et al. (2018) [10]	Case series (n.10)	6–67	Seizure (n.9), breathing difficulties (n.1)	Neurofilament/SMI32, MAP2, Syn, OTX1, TBR1, SOX2, MAP1b, CD34, GFAPd, OLIG2, SMI94/SUFU, EZH2	Temporal (n.9), occipital (n.1)	Appearance: multinodularity Signal: T2w/FLAIR hyperintensity	No recurrence after surgery (n.9); (10–168)
Gonzalez-Quarante et al. (2018) [17]	Case report (n.1)	57	Seizure	Syn, nestin, FGP 9.5, SOX10, OLIG2, p16, ATRX	Temporal	Signal: T2w/FLAIR hyperintensity, no enhancement	No recurrence after surgery (n.1); (12)
Lobo et al. (2018) [18]	Case report (n.1)	19	Vague neurological complaints	NA	Occipital	Appearance: cystic nodules Signal: T2w/FLAIR hyperintensity, no enhancement	NA (no surgery)
Makrakis et al. (2018) [19]	Case report (n.1)	22	Seizures	NA	Parietal	Appearance: small well defined nodules Signal: T2w/FLAIR hyperintensity, T1w hypointensity, no enhancement, slight increased CBV	S (n.1); (6)
Zahra et al. (2018) [20]	Case report (n.1)	33	No relevant symptom	NA	Occipital lobe	Appearance: small nodules Signal: T2w/FLAIR hyperintensity, no enhancement	S (n.1); (NA)
Shitara et al. (2018) [21]	Case report (n.1)	60	No relevant symptom	α-INA, HuC/HuD, Syn, Olig2, NeuN	Frontal	Appearance: multinodular Signal: T2w/FLAIR hyperintensity, T1w hypointensity, no enhancement	No recurrence after surgery (n.1); (16)
Kapucu et al. (2018) [12]	Case report (n.1)	34	No relevant symptom	NA	Parieto-occipital	Appearance: multinodular Signal: T2w/FLAIR hyperintensity, no enhancement	NA
Choi et al.(2019) [8]	Original research (n.7)	10–56	Seizure (n.4), headache (n.3)	α-INA (n.5), Syn (n.7), Olig2 (n.7), MAP2 (n.7), FGFR2 (n.1)	Temporal (n.5), frontal (n.2)	Appearance: solid (n.4), solido-cystic (n.3) Signal: T2w/FLAIR hyperintensity, T1w iso/hypointensity, partial enhancement (n.1)	No recurrence after surgery (n.7); (24–72)
Kodama et al. (2019) [22]	Case report (n.1)	33	Epilepsy	NA	Temporal	Appearance: multinodularity Signal: T2-w/FLAIR hyperintensity, no enhancement	S (n.1); (NA)
Baščarević et al. (2019) [23]	Case report (n.1)	48	Epilepsy	Syn, chromogranin, MAP2, CD34, CD68	Temporal	Appearance: multinodularity Signal: T1w hypointensity, T2w/FLAIR hyperintensity, no enhancement	No recurrence after surgery (n.1); (24)
Nunes Dias et al. (2020) [24]	Case report (n.1)	10	Headache and epilepsy	Syn, Olig2	Temporal	Signal: T2w/FLAIR hyperintensity, loss of gray-white matter differentiation	No recurrence after surgery (n.1); (24)
Gökçe et al. (2020) [25]	Case report (n.2)	27, 21	Headache (n.1), Epilepsy (n.1)	NA	Occipital (n.1), frontal (n.1)	Appearance: small well defined nodules Signal: T2w/FLAIR hyperintensity, T1w hypointensity, no enhancement, no increased CBV	S (n.2); (24–36)
Buffa et al. (2020) [26]	Case series (n.16)	16–77	Seizure (n.5), headache, cognitive impairment (n.3)	NA	Temporal (n.1), frontal (n.8), parietal (n.6), occipital (n.1)	Appearance: small nodules Signal: T2w/FLAIR hyperintensity, T1w iso/hypointensity, no enhancement	S (n.7), NA (n.9); (5–117)
Turner et al. (2020) [27]	Case report (n.1)	5	Cerebral palsy	Olig2	Basal ganglia	Signal: T1w hypointensity, T2w/FLAIR hyperintensity, no enhancement	NA
Lecler et al. (2020) [28]	Original research (n.64)	44.2 ± 15.1	Seizure (n.4), headache (n.45), asymptomatic (n.15)	NA	Temporal (n.13), frontal (n.20), parietal (n.26), occipital (n.5)	Appearance: small nodules; cortical involvement (n.5). Signal: T2w/FLAIR hyperintensity (dot sign n.36), T1w iso/hyper/hypointensity; no restriction on DWI, no increased perfusion, no enhancement	S (n.62), no recurrence after surgery (n.2); (24)
Alizada et al. (2020) [29]	Case series (n.2)	58, 17	Severe headache (n.1), seizure (n.1)	Syn, Olig2, MAP2, GFAP, CD34	Temporal (n.1), frontal (n.1),	Appearance: small nodules Signal: T2w/FLAIR hyperintensity; T1w hypointensity; no enhancement	No recurrence after surgery (n.2); (3–9)
Tan at al. (2021) [30]	Case report (n.1)	7	Sudden activity arrest	NA	Parietal	Appearance: small nodules Signal: T2w/FLAIR hyperintensity; T1w hypointensity	S (n.7); (NA)
Arbuiso et al. (2021) [31]	Case report (n.1)	25	Headache	NA	Parietal	Appearance: solido-cystic Signal: T2w/FLAIR hyperintensity	S (n.1); (12)
Turan et al. (2021) [32]	Original research (n.4)	40–52	No neurologic symptoms	NA	Parietal (n.3), temporal (n.1)	Appearance: multinodularity Signal: T1w hypo-isointensity, T2w/FLAIR hyperintensity, no enhancement, slight decreased perfusion, increased Cho/Cr, decreased Cho/NAA	S (n.4); (12–36)
Bagatto et al. (2021) [33]	Case report (n.1)	45	Seizures	GFAP, Syn, Olig2, ATRX	Parietal	Appearance: multinodularity Signal: T1w isointensity, T2w/FLAIR hyperintensity, no enhancement, decreased CBV, increased Cho/NAA	No recurrence after surgery (n.1); (88)
Sirbu et al. (2022) [13]	Case report (n.1)	29	Seizures	NA	Temporal	Appearance: small nodules Signal: T1w hypointensity, T2-w/FLAIR hyperintensity(dot sign), increased Cho/NAA	NA
Dogra et al. (2023) [34]	Original research (n.48)	10–76	Headache (n.16), seizures (n.5), dizziness (n.5), no clinical neurological symptoms (n.22)	NA	Frontal (n.17), parietal (n.16), temporal (n.5), cerebellum (n.4), occipital (n.3), more lobes (n.3)	Appearance: Multinodular Signal: T2w/FLAIR hyperintensity; T1w hypointensity, no diffusion restriction, no enhancement	S (n.48); (5–30)
Kishi et al. (2023) [35]	Case report (n.1)	67	Epilepsy	GFAP, Syn, Olig2, ATRX, MGMT	Temporal	Appearance: small nodules Signal: T2w/FLAIR hyperintensity	NA
Makino et al. (2024) [36]	Case series (n.2)	30, 20	Intractable epilepsy (n.1), ataxia (n.1)	NA	Parietal (n.1), frontal (n.1),	Appearance: small nodules Signal: T2w/FLAIR hyperintensity, DWI hyperintensity, ADC isointensity	S (n.2); (12)
Do et al. (2024) [37]	Case report (n.1)	62	Headache	NA	Occipital	Appearance: small nodules Signal: T2w/FLAIR hyperintensity, DWI hyperintensity, ADC isointensity	S (n.1); (12)
Pak et al. (2024) [38]	Original research (n.37)	56 ± 12	Cognitive impairment	NA	Parietal (n.11), frontal (n.14), temporal (9), occipital (3)	Appearance: babble (n.35), cortical involvement (n.4) Signal: T2w/FLAIR, hyperintensity DWI hyperintensity, ADC iso-hyperintensity	S (n.37); (21 ± 35)
Lecler et al. (2019) [15]	Original research (n.11)	22–70	Headache (n.4), dizziness (1), no relevant neurological symptoms (n.6)	NA	Vermis and cerebellar hemisphere (n.5), cerebellum (n.6)	Appearance: small nodules, cortical involvement (n.2) Signal: T2w/FLAIR hyperintensity (dot sign in n.9), T1w hypointensity/isointensity (n.11), enhancement (n.1)	S (n.11); (12–43)
Morassi et al. (2020) [14]	Case series (n.2)	41, 44	Headache (n.1), vertigo (n.1)	NA	Vermis and cerebellar hemisphere (n.2)	Appearance: small nodules, cortical involvement (n.1) Signal: T1w hypointensity, T2w hyperintensity, partial suppression on FLAIR; ADC hyperintensity, no enhancement	S (n.2); (12–36)
Abdelouahhab et al. (2022) [39]	Case report (n.1)	54	Seizures	NA	Vermis and cerebellar hemisphere	Appearance: cystic-like lesion Signal: T2w/FLAIR hyperintensity; T1w hypointensity, no diffusion restriction, no enhancement	S (n.1); (12)
Wang et al. (2023) [40]	Case report (n.1)	52	Insomnia and irrirability	GFAP, CD34, HuC/HuD, MAP2, Syn, Olig2	cerebellar hemisphere	Appearance: multinodularity Signal: T1w hypo-isointensity, T2w/FLAIR hyperintensity, slight enhancement	No recurrence after surgery (n.1); (12)
Agarwal et al. (2019) [41]	Case series (n.3)	23–39	Headache (n.2), vertigo (n.1)	NA	cerebellar hemisphere (n.1), vermis (n.2)	Appearance: multinodularity Signal: T1w, hypointensity, T2w/FLAIR hyperintensity, no enhancement	S (n.3); (12)
On et al. (2024) [42]	Case report (n.1)	60	Epilepsy	Syn, Olig2, MAP2, GFAP, CD34	Thalamus	Appearance: multicystic Signal: T2w/FLAIR hyperintensity, no enhancement	NA

n, number; pts, patients; w, weighted; NA, not available; S, stable without surgery. HuC/HuD, Human antigen C/Human antigen D; α-INA, alpha-internexin; MAP2, Microtubule-Associated Protein 2; GFAP, Glial Fibrillary Acidic Protein; Olig2, Oligodendrocyte Transcription Factor 2; MEK1, Mitogen-Activated Protein Kinase Kinase 1; MAP2, Microtubule-associated protein 2; OTX1, Orthodenticle Homeobox 1; TBR1,T-box Brain 1; SOX2, SRY-Box Transcription Factor 2; SMI94/SUFU, Suppressor of Fused; EZH2, Enhancer of Zeste Homolog 2; ATRX, Alpha Thalassemia/Mental Retardation Syndrome X-Linked; Syn, Synaptophysin.

**Table 2:** Representative immunohistochemical expression profile of MVNT.

Category	Markers
Main neuronal markers	
-Immature	HuC/HuD, α-INA
-Mature	NeuN, neurofilaments, synaptophysin, MAP2
Main glial markers	
-Astroglial	GFAP, GFAPδ
-Oligodendroglial	Olig2, SMI94/MBP
Specific neuronal markers	
Cortical layer markers	TBR1, TBR2, OTX1, N200, MAP1B
Developmental/stem cell markers	CD34, Reelin, PAX6, SOX2, Nestin, DCX, PDGFRβ
Interneuronal markers	Calbindin, Calretinin, Parvalbumin, NPY
Neurodegenerative markers	p62, AT8, APP, Mitochondria

HuC/HuD, Human antigen C/Human antigen D; α-INA, alpha-internexin; NeuN, Neuronal Nuclei; MAP2, Microtubule-Associated Protein 2; GFAP, Glial Fibrillary Acidic Protein; GFAPδ, Glial Fibrillary Acidic Protein Delta Isoform; Olig2, Oligodendrocyte Transcription Factor 2; SMI94/MBP, Myelin Basic Protein; TBR, T-box Brain; OTX1, Orthodenticle Homeobox 1, N200, Neurofilament 200 kDa; MAP 1B, Microtubule-associated protein 1B; CD34, Cluster of Differentiation 34; PAX6, Paired Box Protein Pax-6; SOX2, SRY-Box Transcription Factor 2; DCX, Doublecortin; PDGFRβ, Platelet-Derived Growth Factor Receptor Beta, NPY, Neuropeptide Y; p62, protein 62; AT8, Anti-Tau antibody (phosphorylated at Thr231); APP, Amyloid Precursor Protein.

**Table 3:** Differential diagnosis of MVNT in both supratentorial and infratentorial compartments.

Sovratentorial localization	Age Categories	Similarities with MVNT	Differences with MVNT	Immunohistochemical Findings
DNET	Children and adolescents, rarely in adults	- Predilection for temporal lobes - Multiple nodules with bubble appearance in temporal and frontal lobes - Mixed-signal intensity, with some parts being suppressed and a surrounding bright rim on FLAIR	- Cortical involvement - Calcifications and hemosiderin sometimes present - Scalloping of the inner table	NeuN
FCD IIB	Children, adolescents and young adults	- Predilection for frontal and temporal lobes - Radial band extending from cortical region to lateral ventricle	- No bubbly signal alteration	NeuN, GFAP, SMI-32
VRPS	Adults and less frequently in children	- Temporal lobe - Rarely a bubble-like appearance	- CSF-like signals in all sequences	_
Gangliocytoma/Ganglioglioma	Adolescents and young adults (Gangliocytoma) Children and young adults (Ganglioglioma)	- Predilection for temporal lobes - Cystic component with nodular/multinodular appearance.	- Solid tumors with enhancement and calcifications	BRAF mutation V600 CD34, Syn, EGBs, Rosenthal fibers
LGG (oligodendrogliomas)	Adult	- Predilection for temporal lobes - Cystic component with nodular/multinodular appearance	- Infiltrative component with mass effect	Olig2
Infratentorial localization	Age Categories	Similarities with MVNT	Differences with MVNT	Immunohistochemical findings
RGNT	Young adult	- Cerebellar vermis - Multiple nodules with bubble appearance - Multiple satellite lesions	- Green bell pepper sign after gadolinium enhancement, - Intratumoral hemorrhage, - CSF dissemination	Syn, Olig2, GFAP, S-100
Dysplastic cerebellar gangliocytoma	Young adult	- Unilateral cerebellar hemisphere localization - Rare coalescent ‘cystic’ areas due to pronounced vacuolization	- Well-defined cerebellar mass with striated or gyriform ‘tiger-striped’ pattern	GFAP, S-100, NeuN, Syn
PA	Children and adolescents	- In rare cases multicystic appearance with limited or no enhancement	- Large cystic component with an enhancing mural nodule	GFAP, OLIG2, and S-100
VRPS	Adults	- Vermis and, less frequently, cerebellar hemisphere localization - Bubble-like appearance even if rare	- CSF-like signals in all sequences	_

MVNT, Multinodular and Vacuolating Neuronal Tumors; DNET, Disembryoplastic neuroepithelial tumor; FCD IIB, Focal cortical dysplasia type II; VRPS, Enlarged Virchow-Robin perivascular spaces; LGG, Low-grade gliomas; RGNT, Rosette-forming glioneuronal tumors; PA, Pilocytic astrocytomas; NeuN, Neuronal Nuclei; GFAP, Glial Fibrillary Acidic Protein; Olig2, Oligodendrocyte Transcription Factor 2; Syn, Synaptophysin; EGBs, Ependymal Glial Cells.
